# Soil Region Segmentation and Visual Whiteness Analysis in Cold-Region Rice Seedbeds Based on Improved DAC-UNet

**DOI:** 10.3390/plants15111740

**Published:** 2026-06-04

**Authors:** Jiaxin Gao, Feng Tan, Fangming Tian, Zihan Zhu, Yaxuan Wang, Xue Chen, Chengye Yu, Xunpeng Shan

**Affiliations:** 1 College of Engineering, Heilongjiang Bayi Agricultural University, Daqing 163319, China; a441380540@163.com (J.G.); c2723176594@163.com (X.C.); 2 College of Information and Electrical Engineering, Heilongjiang Bayi Agricultural University, Daqing 163319, China; byndtfm@163.com (F.T.); 15707294952@163.com (C.Y.); shanxunpeng@163.com (X.S.); 3 Information Network Center, Panjin Vocational Technical College, Panjin 124000, China; zhu_110121@163.com; 4 College of Civil Engineering and Water Conservancy, Heilongjiang Bayi Agricultural University, Daqing 163319, China; wangyaxuan1980@163.com

**Keywords:** rice seedbed, DAC-UNet, deformable convolution, image processing, Whiteness Index

## Abstract

Soil whitening in cold-region rice seedbeds is visually associated with surface drying and moisture variation. The timely and objective monitoring of soil surface conditions is therefore important for seedbed management. In response to the inefficiencies of manual scouting and the limitations of conventional threshold-based methods under varying illumination and complex soil textures, this study presents a seedbed soil whitening analysis method that combines an enhanced DAC-UNet for semantic segmentation with colour feature analysis. First, a binary segmentation dataset of soil and background was created using RGB seedbed images. Within the U-Net framework, deformable convolution, ASPP++ multi-scale feature aggregation, and the CBAM attention mechanism were introduced to improve the model’s representation of irregular boundaries, scale variations, and complex illumination conditions. Comparative experiments demonstrated that the proposed model achieves 90.63% MIoU, 94.82% mPA, and 97.52% accuracy on the soil segmentation task. Based on the segmented soil region, a Whiteness Index (WI) was formulated to characterize soil surface whitening and generate whitening heatmaps. This enables quantitative description and spatial visualization of whitening characteristics within the soil region. Experimental results showed that the proposed method can effectively capture visual differences among different soil whitening states and provide intuitive visual reference information for soil surface condition analysis in cold-region rice seedbeds.

## 1. Introduction

Rice is one of the most important staple crops worldwide [1], and the vigour of seedlings is a key factor in determining final yield and grain quality. In cold regions such as north-east China, rice seedlings are commonly raised in greenhouse tunnels to maintain suitable temperatures and humidity levels. However, in early spring climatic fluctuations, low temperatures accompanied by strong evaporation and improper irrigation often cause the soil surface to become pale due to moisture loss, salt accumulation, and excessive light reflection—a phenomenon known as ‘whitening’. Not only does seedbed whitening alter the local microclimate, it also indicates insufficient soil water early on. As the topsoil dries and reflectance increases, the amount of water available to plants in the root zone decreases and nutrient uptake is inhibited, leading to stunted growth and decreased seedling vigour. Therefore, accurately detecting and quantitatively analysing seedbed whitening during the seedling stage is of great significance for regulating precision irrigation and diagnosing water–salt stress early on in cold-region rice production.

In recent years, research into monitoring crop growth and assessing soil conditions has advanced rapidly. Traditional approaches to seedbed whitening detection rely primarily on manual observation or threshold-based colour analysis, such as brightness indices, chromatic difference thresholds, or vegetation index calculations. While these methods can be effective under controlled conditions, they are highly sensitive to shadows, specular reflections, and variability in soil texture under complex field illumination, which makes achieving accurate and stable automated recognition difficult. However, with the development of artificial intelligence and agricultural informatics, deep learning-based image segmentation [2] has become widely used in agriculture for tasks such as identifying crop diseases and pests [3,4], detecting weeds [5], counting crops [6], and segmenting field canopies.

Driven by increasing demands for intelligent agriculture and precision management, image-processing and deep-learning methods for rice and soil phenotyping have also developed rapidly. In rice phenotypic analysis, Ramachandran and K.S [6] proposed a lightweight cross-attention-based miniature cross network (TinyCCNET), which significantly reduces computational overhead while maintaining high segmentation accuracy, providing an efficient and reliable solution for UAV-based crop phenotyping. Li et al. [7] improved the semantic segmentation accuracy of small targets in densely adhered rice images through multi-view feature fusion and super-resolution reconstruction, offering an effective approach for rice quality inspection and grain extraction. Hong et al. [7] combined an improved Mask R-CNN with Otsu preprocessing to achieve rice panicle detection and segmentation in open fields, with mean panicle-count error and area error of 16.73% and 3.90%, respectively, supporting rice growth monitoring and yield estimation. Patel and Sharaff [8] leveraged more than 15,000 labelled images and neural-network semantic segmentation for rice variety identification and yield prediction, enabling automated and rapid classification and spikelet/grain counting. For rice disease detection, Zhao et al. [4] proposed an LVR method that fuses language and visual features and incorporates multi-scale attention and efficient feature-extraction modules to achieve accurate and fast segmentation in complex field environments; Samia Yousafzai et al. [9] improved leaf-disease recognition accuracy via transfer learning and optimization algorithms, reaching 98.87% and 97.54% on two benchmark datasets. In rice lodging and seedling identification, Zhuang and Li [2] developed an improved network combining bilinear-interpolation feature fusion and global attention, achieving an MIoU of 88.10% for lodging segmentation and an angle error of 5.364°; Yang et al. [10] proposed the RSHRNet model based on an improved HRNet, integrating an OCR module and coordinate attention to achieve high-accuracy seedling recognition in UAV imagery. For UAV-based rice panicle segmentation and disease detection, Hayat et al. [11] proposed an unsupervised Bayesian approach that outperformed conventional supervised learning in accuracy and recall; Dai et al. [3] developed an AISOA-SSformer model optimized by an annealing-integrated sparrow-search algorithm to achieve high-accuracy leaf-disease segmentation; Xiao et al. [12] proposed WSUNet, which improves CPU inference speed while maintaining accuracy through weighted skip-connection feature fusion; Li et al. [13] presented MMPC-DeepLabv3+, reducing computational cost and parameter count by approximately 93% while improving MIoU, making it suitable for mobile deployment. Gao et al. [14] combined simulated images with a CycleGAN-based domain-adaptive self-supervised segmentation strategy to reduce annotation cost and achieve accurate green-fraction estimation; Ye et al. [15] proposed SY-net, achieving 90.71% mPA for automated rice seed quality inspection, providing a technical basis for high-accuracy and high-efficiency detection. In weed recognition and green-coverage estimation, Kamath et al. [16] evaluated SegNet, PSPNet, and U-Net for classifying different weeds in rice fields, supporting site-specific weed management and precision spraying; Hernández-Hernández et al. [17] developed software that automatically selects the optimal colour space for green-coverage estimation, offering a general tool for agricultural applications.

For soil-image analysis and segmentation, Bai et al. [18] proposed a novel segmentation method based on a dual-scale attention residual module (DARM-UNet), improving segmentation accuracy under uneven staining and blurred boundaries; Zeng et al. [19] reported that the SWFCM algorithm improved purple-soil image segmentation by 6.64–8.25% compared with other methods; Ma et al. [20] combined HSL and CIELAB colour-space features with local blur features to achieve high-accuracy segmentation of soil images in complex field scenes; Yang et al. [21] utilized multi-source remote-sensing data and deep-learning models such as U-Net for automated identification and segmentation of saline soils at the regional scale. Notably, Yida Chen [22] was among the first to apply deep learning to outdoor soil image segmentation, achieving high-accuracy and real-time segmentation using Mask R-CNN and laying a foundation for subsequent soil phenotyping; Onyango and Marchant [23] proposed automatic separation of vegetation and soil pixels in RGB space based on a two-colour reflectance model and illumination-vector analysis. Overall, substantial progress has been made in crop phenotyping, disease recognition, lodging and seedling monitoring, weed classification, and soil image segmentation. However, for soil whitening in cold-region rice seedbeds—especially high-accuracy segmentation and colour-feature analysis under complex backgrounds—there remains a research gap. In particular, encoder–decoder architectures represented by U-Net have shown strong performance in agricultural visual perception due to their ability to learn spatial features and perform pixel-level segmentation. Nevertheless, standard U-Net still suffers from insufficient feature extraction, unclear edge delineation, and limited robustness when facing strong soil specular reflection, complex textures, and blurred whitening boundaries.

Therefore, this study proposes a soil whitening detection method for cold region rice seedbeds. This method is based on an improved DAC-UNet and integrates deep learning segmentation with colour space analysis. This combination enables accurate soil-region segmentation and visual characterization of whitening distribution in complex field scenes. It also provides a technical framework for the visual analysis of the whitening phenomenon on the soil surface of the rice seedling beds. The proposed model incorporates deformable convolution, CBAM, and ASPP++ to enhance multi-scale feature representation and boundary delineation. The combination of these modules enables more stable and accurate segmentation of seedbed soil under challenging illumination and weak texture conditions, providing a reliable basis for subsequent detection of the degree of whitening.

## 2. Materials and Methods

### 2.1. Dataset Establishment

#### 2.1.1. Data Collection

The study was conducted at the Beidahuang Xu Yirong High-Tech Rice Demonstration Park in Hulin City, Jixi, Heilongjiang Province, China. The park’s centre has geographic coordinates of 132°42′51″ E and 45°37′27″ N and an elevation of approximately 79.7 metres.

**Figure 1:**
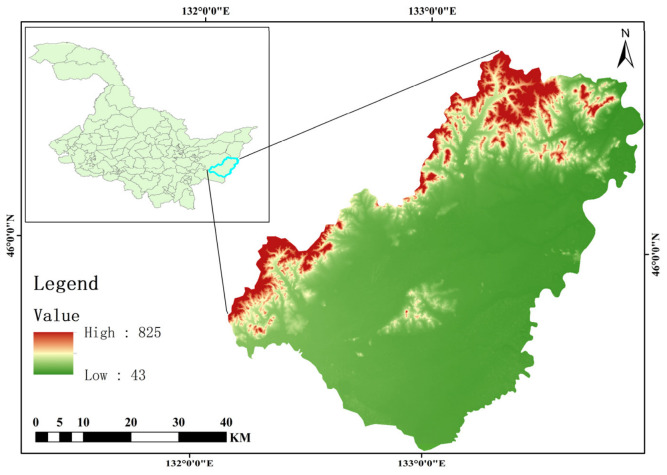
Experimental area.

The experimental area is located within a mid-temperate continental monsoon climate zone. The experimental base is shown in Figure 1. The rice experimental site comprises eight greenhouse tunnels, each measuring 60 m × 12 m, and four rice cultivars were used in this experiment: Longjing 31, Xingjing 10, Kenchuan 102, and Longken 2021. These cultivars are suitable for transplanting or direct seeding in the third accumulated-temperature zone of Heilongjiang Province, China. Image data were collected from 12 April to 18 May 2024 using a Xiaomi 13 smartphone equipped with a CMOS sensor (sourced from Sony Corporation, Tokyo, Japan; maximum aperture: f/5.6) and a 54-megapixel camera. To ensure consistent sampling, 78 fixed sampling points were established in the field, and images were captured daily at the same locations. The camera was positioned at a 45° oblique angle relative to the ground and approximately 50 cm above the seedbed surface, and images were acquired at a maximum resolution of 4608 × 3072 pixels. Data collection was performed once per day between 11:00 and 13:00 to maintain stable lighting conditions, with all images obtained under natural lighting.

#### 2.1.2. Data Annotation and Partitioning

**Figure 2:**
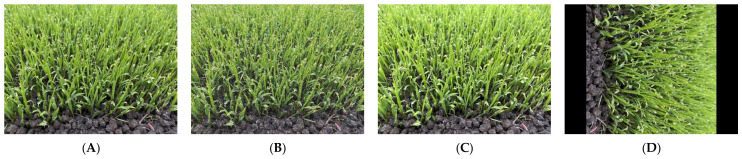
Data enhancement examples: (**A**) original image, (**B**) Gaussian noise, (**C**) random brightness, and (**D**) random rotation.

In order to create a high-quality dataset for soil segmentation in cold-region rice seedbeds, 800 raw soil images were initially collected and curated. Due to significant variations in illumination under natural greenhouse conditions, pronounced differences in soil moisture, and complex backgrounds, the raw images exhibit considerable diversity. To improve the robustness and generalisation of the model, the following data augmentation strategies were applied to the initial dataset: (1) the injection of Gaussian noise to simulate sensor signal perturbations under low-light or high-humidity conditions, (2) random brightness adjustment to mimic differences in imaging across time periods and illumination intensities, and (3) random rotation to account for orientation changes caused by variations in shooting angles. These augmentations effectively reproduce diverse field-related factors, thereby enhancing the model’s adaptability and stability in complex natural scenarios. Examples of the augmented images are shown in Figure 2.

**Figure 3:**
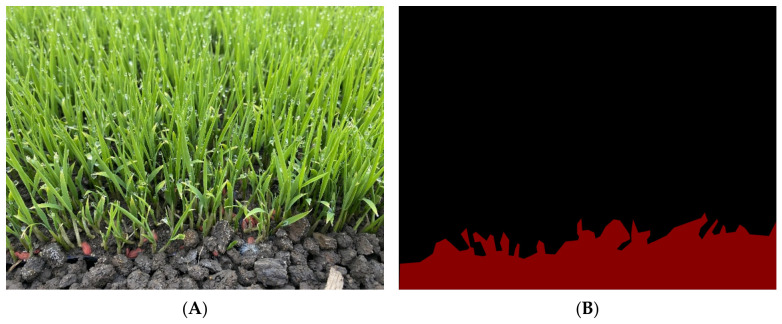
Annotation example: (**A**) original image, and (**B**) labelled image.

Following data partitioning, augmentation was applied only to the training set to avoid data leakage. The original 800 images were first split into training, validation, and test subsets at a ratio of 8:1:1 [24]. Specifically, the training set contains 640 original images for model learning, while the validation and test sets each include 80 original images for performance evaluation and generalisation assessment. After splitting, data augmentation was performed only on the training set, resulting in an expanded training dataset. To ensure accurate annotation, the open-source interactive labelling tool LabelMe [25] was used to generate pixel-wise labels. During annotation, soil regions were assigned to the ‘soil’ class, while all non-soil regions were labelled as ‘background’. Examples of the annotation are provided in Figure 3, and the labelling results were saved in JSON format to ensure good readability and extensibility for subsequent data parsing and model training. This dataset provides a solid foundation for training and optimising the proposed deep learning models.

### 2.2. Improved U-Net Model

#### 2.2.1. Deformable Convolution Module

**Figure 4:**
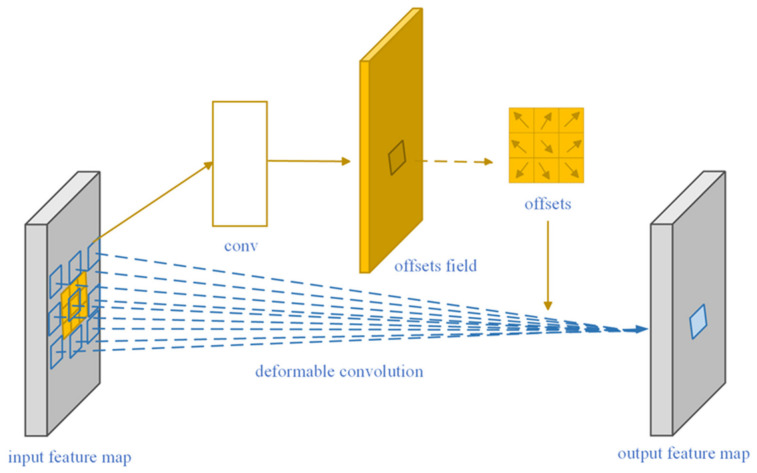
Deformable convolution.

The soil regions in rice seedbeds are highly irregular in shape. Due to variations in seedling density, plant height, leaf expansion, and spatial distribution, the visible soil area between seedlings can vary significantly in terms of both size and shape. These boundaries are often uneven and blurred and are subject to complex deformation. Conventional convolutions use a fixed, regular sampling grid for feature extraction, which limits their ability to model local geometric variations in such irregular regions. Therefore, deformable convolutions, as introduced by Dai, et al. [26] and shown in Figure 4, were incorporated into the high-level encoder convolutional units C4 and C5. This enables adaptive offsets of the sampling locations according to the geometric structure of the input features. It also enhances the network’s sensitivity to complex spatial morphologies. For a standard convolution, the response at position $p_{0}$ on the output feature map can be written as follows:

(1) $$y\left(p_{0}\right)=\sum_{p_{n}\in R}w\left(p_{n}\right)\cdot x\left(p_{0}+p_{n}\right)$$

where R denotes the fixed convolutional sampling region, $w\left(p_{n}\right)$ represents the kernel weight at location $p_{n}$, and $x\left(p_{0}+p_{n}\right)$ is the input feature value at the corresponding position.

In deformable convolution, a learnable offset $\Delta p_{n}$ is introduced for each sampling location, and the output becomes the following:

(2) $$y\left(p_{0}\right)=\sum_{p_{n}\in R}w\left(p_{n}\right)\cdot x\left(p_{0}+p_{n}+\Delta p_{n}\right)$$

**Figure 5:**
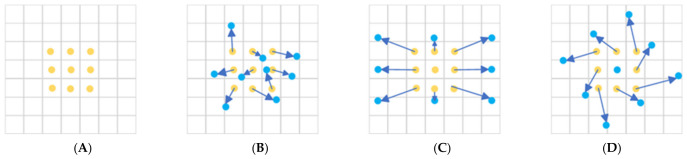
Offset form of deformable convolution: (**A**) original, (**B**) deformation, (**C**) scaling, and (**D**) rotation.

The offsets $\Delta p_{n}$ are learned via an independent offset prediction branch implemented with standard convolutional layers. These offsets allow flexible adjustment of sampling points, enabling the network to focus on actual soil boundaries and texture patterns rather than being constrained by a regular grid. As illustrated in Figure 5, the offsets can represent various geometric transformations, including deformation, scaling, and rotation. Moreover, to preserve feature-map continuity, bilinear interpolation is adopted for non-integer sampling locations:

(3) $$x\left(p_{0}+p_{n}+\Delta p_{n}\right)=\sum_{q}G\left(q,p_{0}+p_{n}+\Delta p_{n}\right)\cdot x\left(q\right)$$

where *G* denotes the interpolation weight. Through this dynamic receptive-field mechanism, the network can adaptively capture soil boundary structures of varying shapes under complex field conditions, substantially improving the geometric flexibility of feature representation.

#### 2.2.2. CBAM Attention Mechanism

In complex field images, variations in brightness, shadows, and the presence of weeds between rice rows can interfere with the discrimination of seedbed soil regions. To enhance the network’s ability to perceive informative regions, a Convolutional Block Attention Module [27] was embedded into the skip connections of the U-Net architecture. By sequentially applying channel attention and spatial attention, CBAM guides the feature fusion to focus on seedbed soil regions and suppresses responses to non-target noise.

Given an input feature map $F\in R^{H\times W\times C}$, CBAM first performs channel attention modeling:

(4) $$M_{c}\left(F\right)=\sigma \left(\mathrm{MLP}\left(\mathrm{AvgPool}\left(F\right)\right)+\mathrm{MLP}\left(\mathrm{MaxPool}\left(F\right)\right)\right)$$

where σ denotes the Sigmoid activation function, AvgPool and MaxPool represent global average pooling and global max pooling, respectively, and MLP is a two-layer fully connected network with shared weights. The resulting channel attention map $M_{c}\left(F\right)$ reweights semantic channels so that the network emphasizes features that are most relevant to the seedbed soil regions.

**Figure 6:**
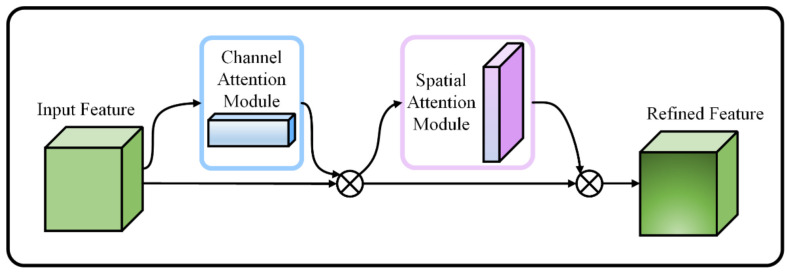
CBAM structure diagram.

Subsequently, spatial attention is applied to the channel-refined features:

(5) $$M_{s}\left(F\right)=\sigma \left(f^{7\times 7}\left(\left[{\mathrm{AvgPool}}_{c}\left(F\right);{\mathrm{MaxPool}}_{c}\left(F\right)\right]\right)\right)$$

where [;] denotes concatenation along the channel dimension, ${AvgPool}_{c}\;$ and ${MaxPool}_{c}\;$ are channel-wise pooling operations, and $f^{7\times 7}$ indicates a 7×7 convolution. The final refined output is computed as follows:

(6) $$F^{\prime }=M_{s}\left(F⊗M_{c}\left(F\right)\right)⊗F$$

where ⊗ denotes element-wise multiplication. Through this two-stage attention mechanism, the network can spatially focus on high-response regions corresponding to the structural patterns of seedbed soil, while strengthening soil texture representations at the channel level. Embedding CBAM within skip connections allows low-level detailed features to be reweighted during fusion, preserving boundary information while suppressing background interference. Consequently, the proposed design markedly improves soil segmentation accuracy under complex illumination conditions in field environments. The CBAM architecture is illustrated in Figure 6.

#### 2.2.3. ASPP++ Module

The seedbed soil regions in rice nurseries exhibit substantial scale variation, ranging from inter-seedling soil patches of only a few centimetres to continuous soil areas spanning more than ten centimetres. Such large-scale disparities make it difficult for convolutions with a single receptive field to simultaneously capture both global context and fine local details. To address this issue, an improved multi-scale feature pyramid module, ASPP++, was incorporated into the bottleneck layer of U-Net. Built upon the conventional ASPP, ASPP++ further integrates deformable convolution and dynamic kernels to enable adaptive fusion of multi-scale features.

Given an input feature map $F\in R^{H\times W\times C}$, the ASPP++ module extracts multi-scale responses using convolutions with different dilation rates $r_{i}$:

(7) $$F_{i}=F_{r_{i}}\left(F\right)=\sum_{p_{n}\in R}w_{i}\left(p_{n}\right)\cdot F\left(p_{0}+p_{n}+\Delta p_{n}^{\left(r_{i}\right)}\right)$$

where $\Delta p_{n}^{\left(r_{i}\right)}$ denotes the adaptively learned offsets under dilation rate $r_{i}$, and $F_{r_{i}}$ represents the offset-enabled dilated convolution operation. The multi-scale features are then fused via channel attention to obtain the final output:

(8) $$Y=\sum_{i=1}^{N}\alpha_{i}\cdot F_{i}$$

In Equation (8), N denotes the number of multi-scale branches corresponding to different dilation rates $r_{i}$, $F_{i}$ represents the output feature map produced by the i-th branch as defined in Equation (7), and $\alpha_{i}$ is the attention weight learned for the i-th scale to adaptively control its contribution during multi-scale feature fusion.

**Figure 7:**
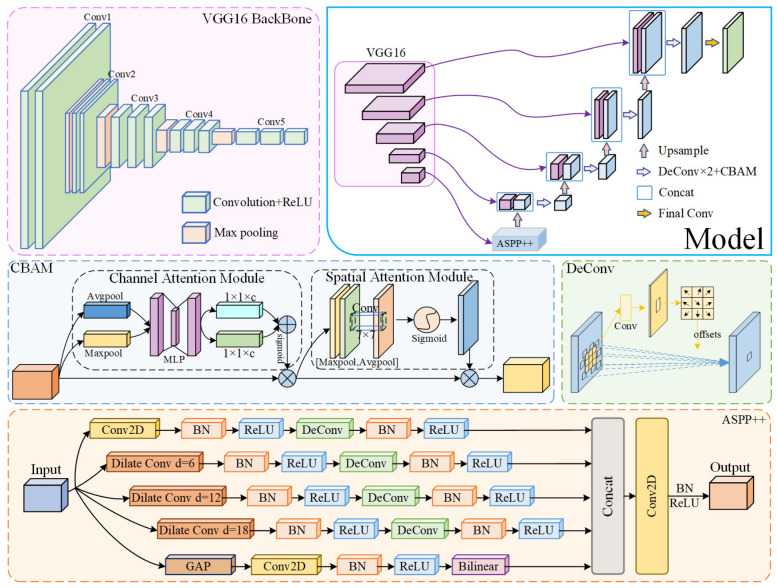
Structure diagram of DAC-UNet.

Through this dynamic multi-scale fusion mechanism, ASPP++ can jointly characterize soil texture fluctuations and structural variations across different scales, thereby improving the representation of both the overall soil contour and boundary details and enabling scale-adaptive responses for seedbed soil segmentation. Structurally, ASPP++ employs parallel dilated-convolution branches together with a 1×1 convolutional global-context branch for collaborative modelling. Specifically, the multi-dilation branches enlarge the receptive field to better capture cross-scale morphology and spatial continuity of soil regions, whereas the global-context branch supplements holistic semantic cues and background priors, which helps suppress interference from rice seedlings, shadows, and weeds. After feature fusion, local boundary details are preserved while global consistency of soil regions is strengthened, providing more stable and informative multi-scale semantic features for the subsequent decoding stage and ultimately improving the accuracy and robustness of seedbed soil segmentation. The overall architecture of DAC-UNet is illustrated in Figure 7.

### 2.3. Soil Whiteness

To objectively evaluate the soil whitening degree in rice seedbeds, this study proposes a mask-constrained Whiteness Index (WI) quantification and heatmap visualization method. Compared with conventional colour-based indices that are calculated over the entire image, the proposed method integrates semantic segmentation with pixel-level colour analysis to restrict the WI computation strictly to soil regions. Using RGB images as input, a soil mask obtained from the semantic segmentation model is first applied to remove non-soil pixels such as rice seedlings, trays, and background structures. By limiting the statistical analysis to soil pixels only, the influence of irrelevant objects and background clutter is significantly reduced, which improves the robustness of the whitening quantification under complex field conditions. In addition, this mask-constrained strategy helps mitigate the impact of illumination variation and specular reflections that frequently occur in greenhouse environments.

First, the RGB channels of the original seedbed images are separated. Let the red, green, and blue channel values at pixel $\left(x,y\right)$ be $R\left(x,y\right)$, $G\left(x,y\right)$, and $B\left(x,y\right)$, respectively, with each value in the range $\left[0,255\right]$. Since dried and whitened soil typically exhibits increased overall brightness accompanied by reduced inter-channel colour differences, the WI is defined as follows:

(9) $$WI\left(x,y\right)=\frac{R\left(x,y\right)+G\left(x,y\right)+B\left(x,y\right)}{3}-|R\left(x,y\right)-G\left(x,y\right)|-|R\left(x,y\right)-B\left(x,y\right)|-|G\left(x,y\right)-B\left(x,y\right)|$$

where $\frac{R\left(x,y\right)+G\left(x,y\right)+B\left(x,y\right)}{3}$ denotes the mean luminance component, and $|R\left(x,y\right)-G\left(x,y\right)|$, $|R\left(x,y\right)-B\left(x,y\right)|$, and ∣G(x,y)−B(x,y)∣ are colour-difference suppression terms. These penalties are introduced to suppress non-soil regions or abnormal specular highlights that may be bright but exhibit strong colour bias. Thus, WI jointly captures both brightness elevation and colour convergence.

Next, the WI computation is constrained to the soil region. Let the soil mask be $M\left(x,y\right)\epsilon \left\{0,1\right\}$, where $M\left(x,y\right)=1$ indicates a soil pixel. The mean WI over the soil-mask region is calculated to represent the overall soil whitening level of an image:

(10) $$\bar{WI}=\frac{\sum_{x,y}WI\left(x,y\right)\cdot M\left(x,y\right)}{\sum_{x,y}M\left(x,y\right)}$$

$\bar{WI}$ reflects the overall visual whitening characteristics of soil within the image and enables quantitative comparison of whitening responses among different samples.

Finally, to visualize the spatial distribution of soil whitening, the pixel-level WI values are min–max normalized and mapped to a pseudo-colour heatmap using the Jet colourmap. This visualization step transforms the scalar WI values into an intuitive colour representation, where warmer colours correspond to higher whitening intensity. The generated heatmap is then alpha-blended with the original RGB image within the soil-mask region, using blending weights of 0.3 for the original image and 0.7 for the heatmap. This fusion strategy preserves the structural details of the original image while emphasizing the spatial variation of the whitening intensity. The value range of the colour bar is determined by $WI_{min}$ and $WI_{max}$, allowing consistent interpretation of whitening levels across different samples. The resulting visualization not only highlights regions with severe whitening but also provides an interpretable basis for spatial analysis of soil moisture stress, thereby providing an intuitive basis for spatial analysis of soil surface whitening characteristics in rice seedbed systems.

## 3. Results and Analysis

### 3.1. Experimental Environment

The experiments were implemented using the PyTorch [28] framework, and the experimental environment is summarised in Table 1. The input image size was set to 256 × 256 pixels. The batch size was set to 16, and the SGD optimiser was adopted with an initial learning rate of 0.01 and a momentum of 0.937. A cosine annealing schedule was used to adjust the learning rate, with a weight decay coefficient of 0.0005. The model was trained for a total of 300 epochs. During training, model checkpoints were saved every 50 epochs on the training set and log files were generated to record loss values on both the training and validation sets. These hyperparameters were carefully selected to facilitate faster convergence, alleviate overfitting, and reduce the risk of the model becoming trapped in local optima.

### 3.2. Model Evaluation Index

The evaluation metrics for semantic segmentation models mainly include accuracy-related indicators represented by mPA and Accuracy, segmentation-performance indicators represented by MIoU, computational-efficiency indicators measured by FPS, and model-complexity indicators quantified by the number of parameters and floating-point operations (FLOPs).

Accuracy metrics can be defined based on the confusion matrix. Taking rice seedbed soil segmentation as an example, soil pixels are treated as the positive class, whereas non-soil regions are treated as the negative class. Accordingly, four outcomes can be obtained: true negatives (TN), false positives (FP), false negatives (FN), and true positives (TP). TP denote soil pixels correctly classified as soil, FP denote background pixels incorrectly classified as soil, FN denote soil pixels incorrectly classified as background, and TN denote background pixels correctly classified as background.

Based on the confusion matrix, the evaluation metrics are calculated as follows:

The mPA calculates the pixel accuracy rate for each category separately, and then takes the average across all categories, as shown in Equation (11). Accuracy represents the proportion of pixels that are paired, as shown in Equation (12).

(11) $$mPA=\frac{1}{k+1}\sum_{i=0}^{k}\frac{TP}{TP+FN}$$

(12) $$Accuracy=\frac{TP+TN}{TP+TN+FP+FN}$$

The intersection-over-union (IoU) measures the overlap between the predicted segmentation region and the ground-truth annotation, defined as the ratio of the intersection area to the union area. The MIoU is the arithmetic mean of IoU across all classes, as shown in Equation (13), where k denotes the number of classes, and k+1 denotes the total number of classes including the background.

(13) $$MIoU=\frac{1}{k+1}\sum_{i=0}^{k}\frac{TP}{FN+FP+TP}$$

In addition, FPS, which stands for frames per second, is adopted to measure the processing speed of the segmentation network. Params refers to the number of trainable parameters expressed in millions, and FLOPs refers to the floating-point operations required for a single forward inference expressed in billions. These two metrics characterize model complexity and computational cost, which are particularly important for deployment in resource-constrained agricultural scenarios.

### 3.3. Ablation Study on Module Combinations

**Figure 8:**
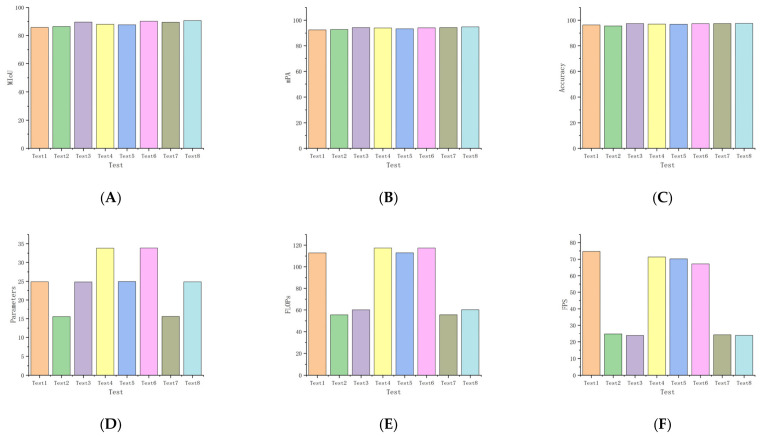
Ablation test result graph. (**A**) MIoU, (**B**) mPA, (**C**) Accuracy, (**D**) Parameters, (**E**) FLOPs, and (**F**) FPS.

Eight ablation experiments were conducted to verify the effectiveness of the proposed modules in improving the performance of the DAC-UNet model for soil segmentation in cold-region rice seedbeds. The experiments focused on three enhancement strategies: deformable convolution, the ASPP++ multi-scale module, and the CBAM attention module. The impact of each strategy on MIoU, mPA, accuracy, parameters, FLOPs, and FPS was evaluated, and the visual comparisons of the modules are shown in Figure 8. The detailed quantitative results for all eight configurations are presented in Table 2.

Experiment 1 used the baseline model without additional modules and achieved an MIoU of 85.81%, an mPA of 92.44%, an accuracy of 96.30%, and an FPS of 74.666, representing the highest inference speed among all configurations. This result indicates that the original U-Net architecture provides strong runtime efficiency and is suitable for real-time scenarios. However, its segmentation consistency and boundary representation capability remain limited under complex illumination and irregular soil-texture conditions, resulting in relatively lower segmentation accuracy. Therefore, although the baseline model offers an excellent speed advantage, its robustness is insufficient for high-precision soil-whitening analysis.

In Experiment 2, deformable convolutions were introduced into the baseline architecture. This improved the MIoU to 86.46% and the mPA to 92.78%, with an accuracy of 95.50%. However, the number of parameters decreased to 15.524 million and FLOPs to 55.474 billion, while FPS dropped markedly to 24.673. This suggests that deformable convolution enhances the modelling capability for irregular and deformed regions. Replacing some of the standard convolution layers reduces the redundant parameters and repeated computations, leading to a decrease in model size and theoretical computational cost. Nevertheless, deformable convolution requires additional offset prediction and feature resampling operations during inference, meaning that the practical runtime does not decrease proportionally with the reduction in FLOPs, resulting in a substantial decrease in FPS. Although this trade-off is acceptable for workstation-based segmentation analysis that prioritises accuracy and robustness, it may limit deployment efficiency on low-power mobile or embedded agricultural devices with constrained computational resources. Therefore, further lightweight optimisation and deployment acceleration strategies should be considered in future work.

Experiment 3 incorporated ASPP++ on top of deformable convolution. The MIoU further increased to 89.53%, while accuracy reached 97.38%, indicating that multi-scale feature aggregation substantially improves segmentation consistency for soil regions with varying textures and illumination conditions. Although FPS slightly decreased to 23.754, the segmentation accuracy improvement was significant compared with Experiment 2. This configuration demonstrates a clear tendency toward accuracy-oriented optimisation, making it suitable for applications where segmentation quality is prioritised over inference speed.

Experiment 4 added ASPP++ to the baseline model without deformable convolution. This configuration achieved an MIoU of 88.04% and maintained a high FPS of 71.379. Compared with the baseline model, segmentation accuracy improved notably while inference speed remained close to real-time performance. Although the parameter count and FLOPs increased, the actual runtime efficiency remained high. These results indicate that ASPP++ provides an effective balance between segmentation accuracy and computational efficiency, particularly for scenarios requiring both robustness and relatively fast inference.

Experiment 5 introduced CBAM into the baseline model. The MIoU improved to 87.66% while FPS remained at 70.250, which is also close to the baseline speed. Compared with Experiment 4, the CBAM-based configuration achieved slightly lower accuracy but required fewer parameters and FLOPs. This suggests that CBAM effectively enhances the attention to important soil regions with relatively low additional computational burden. Therefore, this configuration provides a favourable trade-off between accuracy and efficiency and is suitable for lightweight deployment or real-time agricultural monitoring scenarios.

Experiment 6 combined ASPP++ and CBAM, achieving an MIoU of 90.17%, an mPA of 94.07%, and an accuracy of 97.35%, while maintaining an FPS of 67.088. Compared with the DAC-UNet, this configuration achieved only slightly lower segmentation accuracy (ΔMIoU = 0.46%) but operated at nearly three times the inference speed. This confirms that the combination of ASPP++ and CBAM provides an excellent balance between segmentation performance and runtime efficiency, making it highly attractive for real-time or resource-constrained deployment scenarios. Nevertheless, in this study we selected DAC-UNet as the final model for two primary reasons. First, the main objective of this work was to establish a high-precision segmentation foundation for subsequent soil-whitening analysis, where even small improvements in boundary delineation and region consistency can directly influence the reliability of the pixel-wise Whiteness Index (WI). The deformable convolution module provides adaptive geometric feature extraction, which is particularly beneficial for capturing irregular soil-seedling boundaries and complex local structures under greenhouse conditions. This capability cannot be fully achieved by ASPP++ and CBAM alone. Second, the proposed whitening-analysis framework is mainly intended for offline or semi-offline agronomic assessment rather than strict real-time applications. Therefore, inference speed was not the primary limiting factor in the current study. Although DAC-UNet reduces the FPS to 23.810, this speed remains sufficient for batch processing and practical greenhouse seedbed analysis. Consequently, this study prioritized segmentation precision and robustness over maximum inference throughput, leading to the selection of DAC-UNet as the final configuration.

Experiment 7 combined deformable convolution and CBAM. This configuration achieved an MIoU of 89.45% with relatively low parameters and FLOPs, but FPS remained limited at 24.195 due to the runtime overhead introduced by deformable convolution. Compared with Experiment 6, this configuration provided slightly lower accuracy and significantly lower inference speed. These results suggest that the performance gains introduced by deformable convolution are accompanied by substantial efficiency loss, reducing its suitability for real-time applications despite its improved capability for modelling irregular structures.

Finally, Experiment 8 incorporated all three modules to form the full DAC-UNet model. This configuration achieved the best overall segmentation performance, with an MIoU of 90.63%, an mPA of 94.82%, and an accuracy of 97.52%. However, FPS decreased to 23.810. Compared with Experiment 6, the full model improved MIoU by only 0.46% while the inference speed decreased substantially from 67.088 FPS to 23.810 FPS. Therefore, although the full DAC-UNet provides the highest segmentation accuracy and strongest robustness under complex soil conditions, the improvement in accuracy must be considered together with the associated runtime cost. In practical applications, Experiment 6 may represent a more balanced solution for real-time or embedded agricultural systems, whereas the full DAC-UNet is more suitable for scenarios where segmentation precision is prioritised over inference speed.

Overall, the ablation results demonstrated that different module combinations exhibit distinct trade-offs between segmentation accuracy and computational efficiency. Deformable convolution mainly contributes to improved modelling of irregular soil boundaries but significantly reduces runtime speed. ASPP++ effectively enhances multi-scale feature representation while maintaining relatively high efficiency, and CBAM improves feature discrimination with limited computational overhead. From a Pareto perspective, the ASPP++ + CBAM configuration provides the most balanced compromise between accuracy and inference speed, whereas the full DAC-UNet achieves the highest overall segmentation performance at the cost of reduced runtime efficiency. These results demonstrated the flexibility of the proposed framework for different deployment requirements in agricultural soil segmentation tasks.

### 3.4. Confusion Matrix

The confusion matrix is an important tool for evaluating the classification performance of deep-learning models [29]. It takes the form of a matrix, with the rows representing the true labels and the columns representing the predicted labels. Each entry reflects the number or proportion of samples from a given true class that are assigned to a predicted class. The diagonal elements correspond to correct predictions, while the off-diagonal elements indicate misclassifications. Analysing the confusion matrix enables one to directly identify false-negative and false-positive patterns for each class, providing quantitative evidence for subsequent architectural design and parameter tuning.

**Figure 9:**
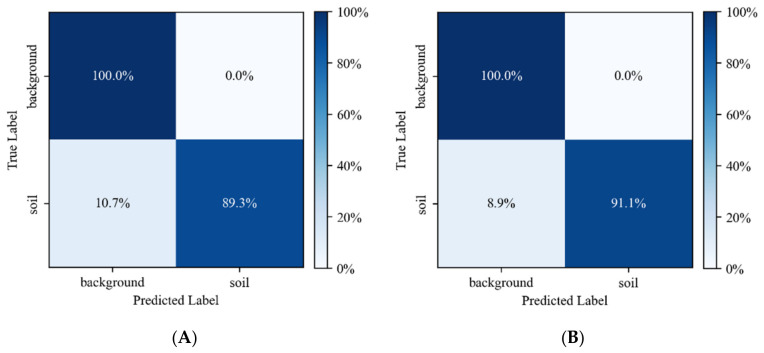
Confusion matrix. (**A**) U-Net, and (**B**) DAC-UNet.

For the binary soil segmentation task in cold-region rice seedbeds, the model’s ability to distinguish between ‘background’ and ‘soil’ pixels is of primary interest. Figure 9A shows the normalised confusion matrix of the baseline U-Net on the test set. As can be seen, all true background pixels are correctly predicted as background, with none misclassified as soil. This indicates extremely high precision for background recognition, with almost no cases of non-soil regions being falsely detected as soil. However, for the soil class, only 89.3% of true soil pixels are correctly predicted as soil, while 10.7% are misclassified as background. This suggests that the baseline model still misses detections in soil regions, particularly in patches with weak texture, brightness similar to the background, or blurred boundaries, where feature representation remains insufficient.

Figure 9B shows the normalised confusion matrix for the improved DAC-UNet model on the same test set. The results show that true background pixels continue to be predicted as background without generating any additional false positives. For the soil class, the correct prediction rate increases from 89.3% to 91.1%, while the proportion misclassified as background decreases from 10.7% to 8.9%. In other words, DAC-UNet reduces the false-negative rate for soil pixels by 1.8 percentage points. These findings suggest that the improved network maintains highly accurate background recognition while further enhancing sensitivity and discriminative capability for soil targets. This enables better separation of soil and non-soil regions with similar brightness and texture.

Overall, DAC-UNet, which incorporates deformable convolution, multi-scale feature extraction, and attention mechanisms, effectively improves segmentation performance for seedbed soil in cold-region rice production. It has a particular advantage in reducing missed soil detections and improving target recall. As the confusion matrices show, DAC-UNet is a better choice than U-Net for the core segmentation model for seedbed soil detection and subsequent identification of the whitening region.

### 3.5. Comparative Test

**Figure 10:**
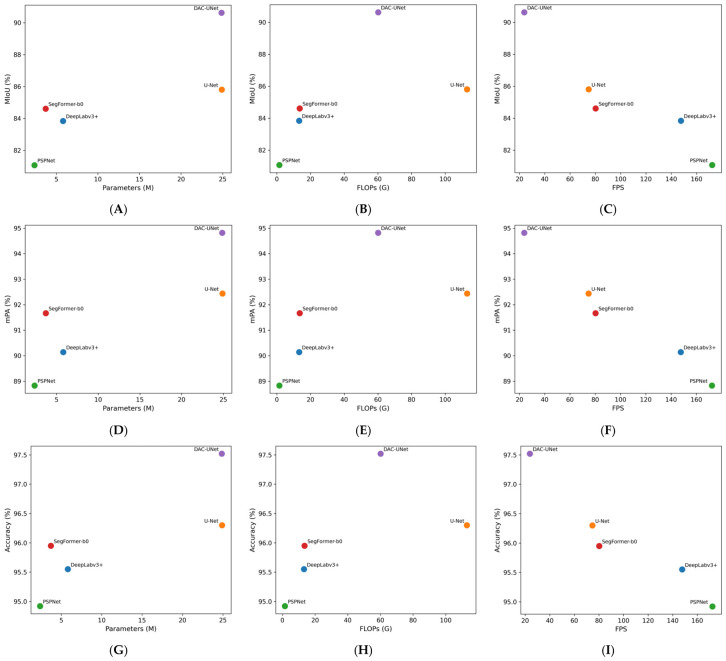
Trade-off plots between performance metrics and efficiency factors across models. (**A**) Trade-off between MIoU and model parameters. (**B**) Trade-off between MIoU and FLOPs. (**C**) Trade-off between MIoU and FPS. (**D**) Trade-off between mPA and model parameters. (**E**) Trade-off between mPA and FLOPs. (**F**) Trade-off between mPA and FPS. (**G**) Trade-off between Accuracy and model parameters. (**H**) Trade-off between Accuracy and FLOPs. (**I**) Trade-off between Accuracy and FPS.

The effectiveness of the improved DAC-UNet model was validated in the soil-whitening segmentation task for cold-region rice seedbeds by comparing it with several widely used semantic segmentation models, including DeepLabv3+ [30], U-Net and PSPNet [31], and the Transformer-based SegFormer-b0 model. The results of the experiments are summarised in Table 3. As shown in the table, the improved DAC-UNet achieved the best overall performance across all major evaluation metrics. Specifically, it attained an MIoU of 90.63%, an mPA of 94.82%, and an accuracy of 97.52%, all of which were higher than the corresponding results of the other compared models.The trade-offs between segmentation accuracy and model efficiency across these models are further illustrated in Figure 10.

Compared with DeepLabv3+, U-Net, PSPNet, and SegFormer-b0, the improved DAC-UNet achieved MIoU improvements of 6.79%, 4.82%, 9.56%, and 6.02%, respectively. Meanwhile, the mPA increased by 4.68%, 2.38%, 5.99%, and 3.15%, respectively, and the accuracy improved by 1.97%, 1.22%, 2.60%, and 1.57%, respectively. These results indicate that DAC-UNet provides stronger feature extraction capability and superior regional consistency in pixel-level soil segmentation tasks.

In terms of model complexity, DAC-UNet contains 24.863 million parameters, which is almost identical to the U-Net baseline, but notably higher than DeepLabv3+, PSPNet, and SegFormer-b0. Regarding computational cost, U-Net requires the largest FLOPs, whereas PSPNet shows the lowest computational complexity among the compared methods. Due to the integration of deformable convolution, CBAM attention, and ASPP++ modules, DAC-UNet shows a reduced inference speed of 23.81 FPS, which is lower than DeepLabv3+, PSPNet, and SegFormer-b0. Nevertheless, the substantial gains in segmentation accuracy and robustness compensate for the decrease in inference speed, making DAC-UNet more suitable for applications where segmentation precision and stability are prioritised.

Overall, within a reasonable computational budget, the improved DAC-UNet demonstrates clear advantages in terms of segmentation accuracy, pixel-level recognition capability, and robustness in soil-whitening regions of cold-region rice seedbeds. These results suggest that it has strong comprehensive performance and practical application potential, providing more reliable support for soil-region segmentation and visual whitening analysis in rice seedbeds.

### 3.6. Visualization of Experimental Results

**Figure 11:**
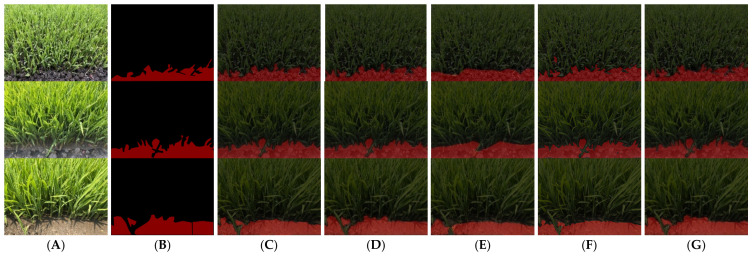
Soil segmentation effect diagrams of different models: (**A**) original image, (**B**) mask, (**C**) DeepLabv3+, (**D**) U-Net, (**E**) PSPNet, (**F**) SegFormer-b0, and (**G**) DAC-UNet.

To account for different illumination conditions and soil states, three representative scenarios were considered in order to evaluate the effectiveness of the models for rice seedbed soil segmentation: (i) low light and moist soil; (ii) normal illumination and relatively dry soil; (iii) strong illumination and slightly dry soil. Figure 11 compares the segmentation results of DeepLabv3+, U-Net, PSPNet, SegFormer-b0, and the proposed DAC-UNet under these three conditions.

Under the low-light and moist-soil condition, both DeepLabv3+ and U-Net can localise the main soil regions. However, they tend to incorrectly include small portions of rice plants near the seedling bases in their predictions, leading to boundary confusion at the soil–seedling interface. PSPNet shows an over-smoothing tendency, producing unnaturally smooth upper soil boundaries and suppressing local details. SegFormer-b0 demonstrates relatively good global region perception and maintains more continuous soil masks than DeepLabv3+ and PSPNet. Nevertheless, it still exhibits slight boundary deviations and local misclassification near dense seedling regions under weak illumination conditions. In contrast, DAC-UNet yields better region connectivity and boundary fluctuations that are more consistent with the real surface morphology of the seedbed. At the junction between seedlings and soil, DAC-UNet neither merges plant pixels into the soil mask nor excessively shrinks the soil region, resulting in a more reasonable overall mask shape.

The normal-illumination and relatively dry-soil condition further highlights differences in robustness. In this case, the soil masks produced by DeepLabv3+ and U-Net exhibit more pronounced boundary instability, with noticeable boundary fluctuations as the illumination and appearance of the soil change. PSPNet continues to demonstrate clear under-segmentation and excessive smoothing, compressing or simplifying soil edges, and introducing more plant pixels into the predicted soil region. SegFormer-b0 achieves relatively stable segmentation results under this condition and preserves overall soil-region continuity well. However, compared with DAC-UNet, its boundary fitting capability remains weaker in regions containing irregular soil textures and local shadow interference. In contrast, DAC-UNet maintains a more stable soil-band width and stronger boundary consistency, demonstrating superior adaptability to contrast the reduction and texture perturbations caused by illumination variation.

Under strong illumination and slightly dry soil conditions, the challenge extends beyond illumination to substantial changes in soil colour distribution and shadow interference. Qualitative visualisation shows that all models exhibit inconsistent boundary shifts under these conditions, with the upper boundary of the soil region tending to shift downwards, causing the predicted soil area to shrink relative to the ground truth. Additionally, plant shadows induced by strong light lead to local contraction of the soil mask. DeepLabv3+ and PSPNet are more sensitive to reflective interference and brightness variation, while U-Net exhibits noticeable boundary fragmentation in local regions. SegFormer-b0 maintains relatively good global consistency under strong illumination but still shows local boundary smoothing and incomplete recovery of irregular soil edges. Although DAC-UNet is also affected to a certain extent, it demonstrates the least degradation among the compared models, providing more stable alignment with the true soil region while preserving higher mask integrity and boundary interpretability.

Overall, DAC-UNet demonstrates superior boundary adherence, region connectivity, and segmentation consistency across different illumination and soil conditions. This is particularly important for the proposed whitening-analysis framework, which combines soil segmentation with subsequent colour-based whitening characterization. Cleaner and more stable soil masks improve the reliability of the subsequent computation of soil whitening distribution and reduce the influence of non-soil interference regions. Therefore, DAC-UNet is better suited to serving as the backbone for soil segmentation in cold-region rice seedbed soil-whitening analysis due to its ability to balance segmentation accuracy and robustness under complex greenhouse environments.

### 3.7. Visualization of Soil Whiteness Heat Map Results

**Figure 12:**
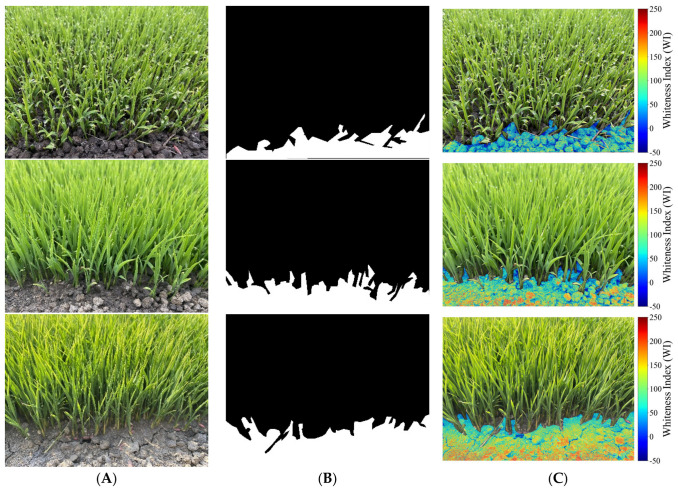
Soil whiteness heat map: (**A**) original image, (**B**) soil mask, (**C**) heat map of soil whitening.

Figure 12 illustrates the proposed visualisation pipeline for the degree of soil whitening. Three representative states were selected: moist soil, partially whitened soil, and largely whitened soil. Figure 12A shows the original seedbed image, Figure 12B shows the soil segmentation mask generated by DAC-UNet, and Figure 12C shows the soil whitening heatmap computed within the soil mask region. Constraining the whitening metric to soil pixels only effectively avoids interference from rice seedlings and background regions, meaning the colour variation in the heatmap reflects the soil’s surface moisture condition rather than the changes in plant brightness or illumination.

Under moist soil conditions, the soil has an overall darker appearance and the heatmap is dominated by cool colours, with only a few areas showing relatively high responses. The corresponding mean Whiteness Index is relatively low, with WI_mean = 41.356, indicating that the soil surface brightness and whitening degree are minimal. The higher localised responses are mainly associated with small soil granule protrusions. Overall, the heatmap is consistent with human visual perception and captures the characteristic low whitening degree of moist soil.

Under the partially whitened condition, the heatmap contains both cool-colour regions and locally clustered warm-colour regions. This suggests that soil whitening does not occur uniformly but is concentrated in specific areas. The corresponding mean Whiteness Index increases to WI_mean = 89.991, reflecting the gradual enhancement of surface whitening. These warm regions typically correspond to drier, brighter locations, whereas the whitening degree near the seedling roots is relatively low. The results show that the heatmap can reflect not only the overall whitening level but also the spatial distribution of the whitening regions.

In this largely whitened state, warm colours dominate the heatmap, and high-response regions expand substantially, indicating a high overall degree of soil whitening. The corresponding mean Whiteness Index further increases to WI_mean = 117.585, demonstrating a pronounced whitening response across the soil surface. Meanwhile, a small number of cool-colour areas remain, mainly in shaded or darker localised regions, reflecting lower whiteness in these areas. Compared with the previous two cases, the high-response areas in this scenario are more continuous and widespread, consistent with large-area soil whitening in practice.

Overall, the three states exhibit a consistent transition pattern. As the soil changes from moist to partially whitened and then extensively whitened, the high-response regions in the heatmap progressively increase and expand, accompanied by a continuous increase in WI_mean values. Constrained by the soil mask, the heatmap stably visualises the spatial distribution of soil whitening within the soil region. This provides an intuitive visual representation of soil surface whitening characteristics under different moisture conditions.

## 4. Discussion

### 4.1. The Performance of the Model

To verify the performance of the improved soil segmentation model for cold-region rice seedbeds in greenhouses, comparative experiments, ablation studies, and qualitative visual analyses were conducted. The results of these experiments indicated that the DAC-UNet model achieves the highest MIoU, mPA, and Accuracy among those evaluated. While its FPS is not the highest, the reduction in inference speed is acceptable. Moreover, the substantial accuracy improvement means that the inference speed still meets real-time requirements.

According to the experimental results, the proposed method achieves an MIoU of 90.63% for rice seedbed soil segmentation. Both the proposed method and the seedling-row segmentation approach based on CAD-UNet, as proposed by Luo et al. [32], introduce CBAM attention and deformable convolution within the U-Net framework and report performance gains, thus supporting the effectiveness of these architectural enhancements. In contrast, the work by Al-Naji et al. [33] relies primarily on mean RGB values within small regions of interest and a shallow neural network to classify soil moisture status discreetly. This study uses an improved DAC-UNet to perform pixel-level segmentation, precisely delineating seedbed soil regions in cold-region rice production. It also combines a soil-whitening heatmap to provide continuous visual characterization of soil surface whitening distribution, making it suitable for intuitive analysis of spatial whitening variation during the rice seedling stage.

### 4.2. Limitations and Improvement Methods

This study presents an improved DAC-UNet model that achieves high accuracy and robustness in soil segmentation in cold-region rice seedbeds. The model demonstrated notable improvements in MIoU, precision, and recall by incorporating deformable convolution, a multi-scale feature pyramid, and CBAM attention modules, effectively mitigating the adverse effects of complex field backgrounds on segmentation performance. Nevertheless, despite its strong overall performance, the model has several limitations, as follows:

(1) In this study, the proposed model was mainly compared with several widely used semantic segmentation networks, including U-Net, PSPNet, DeepLabv3+, and SegFormer-b0. These models represent classic encoder–decoder and multi-scale segmentation frameworks and are commonly used as benchmark methods in many agricultural image segmentation studies. However, with the rapid development of deep learning, some updated architectures and some high-performance CNN-based frameworks have also been proposed. These models have demonstrated strong capabilities in complex scene understanding and dense prediction tasks. Due to the scope of this study and the need to maintain consistent experimental settings, these newer architectures were not included in the comparative experiments. Future work will incorporate more recent seg-mentation models to provide a more comprehensive benchmark evaluation and further investigate the relative advantages of the proposed method in agricultural soil segmentation scenarios.

(2) The dataset used in this study was collected from a representative rice seedling nursery site in the cold region of Heilongjiang Province under relatively consistent acquisition conditions. The dataset reflects the typical soil background and management conditions of the target seedbed environment, which contributes to stable model training and evaluation. Nevertheless, the geographic coverage and environmental diversity of the dataset remain relatively limited. Differences in soil types, cultivation practices, greenhouse structures, and environmental conditions in other regions may lead to variations in image characteristics. Therefore, although the proposed method demonstrates strong performance under the current experimental conditions, further validation under broader scenarios would be beneficial. In particular, when applied to substantially different soil environments, such as tropical soils or high-clay soils, the model may exhibit certain radiometric biases and may require additional fine-tuning or domain adaptation to maintain segmentation robustness. Future studies will expand the dataset by incorporating samples from additional locations and acquisition conditions, thereby enabling a more comprehensive assessment of the robustness and generalization capability of the proposed method. Due to the fixed camera angle and height, the soil-to-vegetation area ratio in the collected dataset is relatively homogeneous, which limits the model’s generalization to extreme scenarios; future work will include images from varying growth stages, planting densities, and seedbed positions to enrich this diversity.

## 5. Conclusions

This study focused on soil whitening in rice seedbeds in cold regions, which is a typical visual phenomenon associated with soil moisture imbalance. An integrated analysis framework combining image segmentation and colour-based whitening analysis was established. The main conclusions are summarised below:

(1) An improved DAC-UNet-based soil segmentation model was proposed. Built upon the U-Net architecture, this model integrates deformable convolution, an ASPP++ multi-scale feature pyramid, and a CBAM attention mechanism, enhancing the representation of irregular boundaries, multi-scale soil regions, and complex textures. Ablation experiments demonstrated that these modules contribute complementary performance improvements. The final model achieved 90.63% MIoU, 94.82% mPA, and 97.52% accuracy on the collected seedbed soil segmentation dataset, outperforming the compared baseline models. These results indicate that the proposed method provides effective and robust soil-region segmentation under complex seedbed conditions.

(2) Based on the segmentation results, a soil whitening quantification method using RGB colour features was developed. By restricting the analysis to segmented soil regions, interference from rice seedlings and background regions was effectively reduced, enabling visualisation and quantitative analysis of soil whitening distribution. Experimental results demonstrated that the proposed framework can effectively characterise spatial variations in soil whitening under different illumination and soil conditions within the experimental dataset.

Overall, the proposed method combining DAC-UNet segmentation and colour-based whitening analysis achieved good performance for soil-region extraction and whitening visualisation in cold-region rice seedbeds. Future work will further validate the method using multi-region and multi-season datasets and investigate its integration with soil moisture sensing and embedded intelligent agricultural systems to improve practical applicability and generalisation capability.

## Figures and Tables

**Table 1:** Test environment.

Category	Environment Parameter
Operating System	Windows 11 64 bits
Development Tool	PyCharm 2024.1.2
CPU	Intel Core i7-14650HX 2.20 GHz
GPU	NVIDIA GeForce RTX 4060
Deep Learning Framework	PyTorch 2.0.1
Scripting Language	Python 3.12
RAM	16.0 GB

**Table 2:** Ablation test results.

Test	Baseline	Deform	ASPP++	CBAM	MioU (%)	mPA (%)	Accuracy (%)	Parameters (M)	FLOPs (G)	FPS
1	✓				85.81	92.44	96.30	24.891	112.918	74.666
2	✓	✓			86.46	92.78	95.50	15.524	55.474	24.673
3	✓	✓	✓		89.53	94.28	97.38	24.819	60.103	23.754
4	✓		✓		88.04	93.95	96.93	33.810	117.353	71.379
5	✓			✓	87.66	93.34	96.85	24.935	112.951	70.250
6	✓		✓	✓	90.17	94.07	97.35	33.854	117.386	67.088
7	✓	✓		✓	89.45	94.25	97.36	15.568	55.507	24.195
8	✓	✓	✓	✓	90.63	94.82	97.52	24.863	60.136	23.810

**Table 3:** Comparison of soil segmentation results of rice seedbeds in cold regions under different models.

Models	MioU (%)	mPA (%)	Accuracy (%)	Parameters (M)	FLOPs (G)	FPS
DeepLabv3+	83.84	90.14	95.55	5.813	13.217	147.672
U-Net	85.81	92.44	96.30	24.891	112.918	74.666
PSPNet	81.07	88.83	94.92	2.376	1.510	172.320
SegFormer-b0	84.61	91.67	95.95	3.715	13.537	80.244
DAC-UNet	90.63	94.82	97.52	24.863	60.136	23.810

## Data Availability

The original contributions presented in this study are included in the article; further inquiries can be directed to the corresponding author.
